# An Isolated Second Carpometacarpal Joint Dislocation Treated With Open Reduction and Transfixation With K-wire: A Rare Case Report

**DOI:** 10.7759/cureus.56338

**Published:** 2024-03-17

**Authors:** Saravanan Alagesan, Silambarasi Nagasamy, Pradeep Elangovan, Haemanath Pandian, Ganesh Anantharaman

**Affiliations:** 1 Orthopaedics, Chettinad Hospital and Research Institute, Chennai, IND

**Keywords:** second cmc joint, k-wire, closed reduction, dorsal dislocation, carpometacarpal joint dislocation

## Abstract

Carpometacarpal (CMC) joint dislocation is a rare injury that can be easily missed due to non-specific clinical signs and radiologic findings. We report a patient with an isolated and irreducible dorsal dislocation of the second CMC joint, which required an open reduction and K-wire transfixation. An extensor tendon was found to be interposed between the base and the trapezoid. K-wire removal was done after three weeks and following the removal of the wire, rehabilitation was started.

## Introduction

Carpometacarpal (CMC) joint dislocation is an uncommon injury that can be easily missed. It represents less than 1% of global hand trauma [1]. Fracture-dislocations of the CMC joints are more frequent than isolated dislocations, of which, dorsal dislocations are more common. These types of complex carpometacarpal injuries are a rare occurrence, with the last case of isolated second CMC joint dislocation reported in 1987 [2]. It is often missed due to non-specific clinical signs and radiologic findings. Also, the second CMC joint is extremely stable, which can be attributed to its strong ligamentous and bony stabilizers [2]. The articular facets, dorsal, volar and intermetacarpal ligaments provide a stable construct for this joint [3]. In an early presentation, a closed reduction may be possible. But in delayed presentations or unstable dislocations, open reduction may be required. We report a case of an isolated and irreducible dorsal dislocation of the second CMC joint, which required an open reduction and K-wire transfixation.

## Case presentation

**Figure 1:**
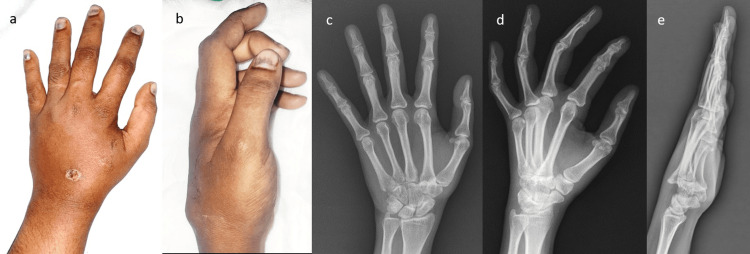
Clinical pictures and X-rays at presentation (a) and (b) clinical pictures showing swelling over the dorsum of the hand, (c) anteroposterior and (d) oblique view of hand showing minimal bony overlap at second carpometacarpal joint, (e) lateral view showing dorsal dislocation at second carpometacarpal joint

**Figure 2:**
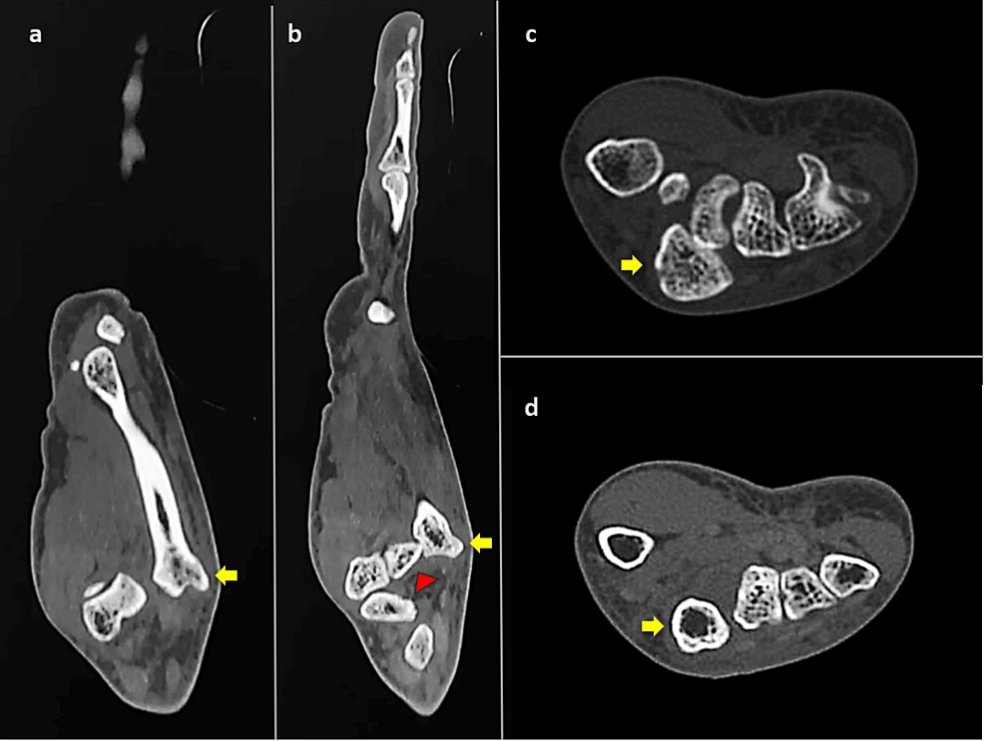
Computed tomography of the hand (a) and (b) sagittal computed tomography sections demonstrating dorsal dislocation of the second metacarpal. Yellow arrow indicates dorsal dislocation of the base of the second metacarpal, while the red arrowhead indicates the trapezoid bone. (c) and (d) axial computed tomography sections showing disruption of the transverse metacarpal arch. Yellow arrow indicates the second metacarpal, which is no longer within the arch.

A 35-year-old female came to the Orthopaedics outpatient department with complaints of pain and swelling over the left hand for last one week. She gave an alleged history of skid and fall from her two-wheeler, thereby landing on an outstretched hand. On clinical examination, there was an abrasion of about 1 X 1 cm over the dorsum of the hand with a bony prominence (Figure 1) and tenderness present over the dorsal aspect of the base of the second metacarpal. She had limitation of active, as well as passive movements of the second metacarpophalangeal joint. Plain radiograph in anteroposterior and oblique view did not show any obvious bony abnormality but the lateral view of left hand showed isolated dorsal dislocation of the second CMC joint (Figure 1). Computed tomography scan was taken for better visualization of the CMC joint which revealed dorsal displacement of second CMC joint without any evidence of fracture (Figure 2).

**Figure 3:**
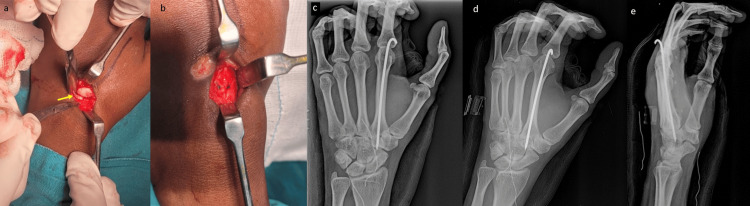
Intra-operative pictures and postoperative X-rays (a) Intra-operative picture showing the base of the second metacarpal with the interposed extensor tendon (arrow) between the base of second metacarpal and the trapezoid, (b) sutured dorsal capsule. Postoperative X-rays showing the reduced second carpometacarpal joint transfixed with a K-wire; (c) anteroposterior, (d) oblique and (e) lateral views

The patient was taken up for closed reduction under supraclavicular block. Closed reduction was found to be unsuccessful. Hence, open reduction was decided upon. Under fluoroscopy guidance, a 5cm long transverse incision was made over the base of the second metacarpal. The dorsal capsule was found to be disrupted. An extensor tendon was found to be interposed between the base of the second metacarpal and the trapezoid (Figure 3). The tendon was retracted and reduction was attempted, which was found to be satisfactory under fluoroscopy. The reduction was unstable and hence, a single 1.5mm K-wire was passed through the second metacarpal and into the trapezoid, stabilizing the second CMC joint by transfixation (Figure 3). Regular wound inspection and dressing was done.

**Figure 4:**
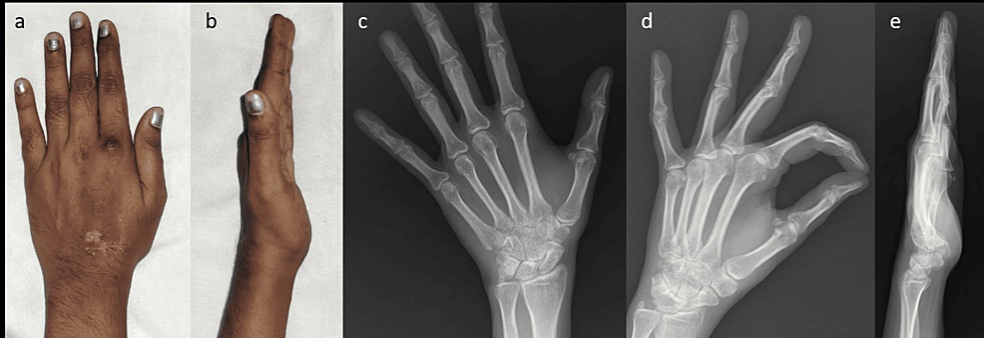
Clinical pictures and X-rays after K-wire removal Clinical pictures post-K-wire removal showing (a) dorsum and (b) lateral aspect of hand, (c) anteroposterior (d) oblique and (e) lateral views after K-wire removal

Suture removal was done on the 12th postoperative day. K-wire removal was done after three weeks (Figure 4) and following the removal of the wire, rehabilitation was started. Patient returned to her routine activities by the end of fourth week.

## Discussion

This case report presents a patient with isolated second CMC joint dislocation, treated successfully with closed reduction and K-wire transfixation. CMC joint dislocations, a subset of complex carpometacarpal injuries, make up less than one of global hand trauma. These occur often due to high-energy trauma, where the direct impact causes dislocation of metacarpal base posteriorly following a flexion force subjected to the metacarpal head. Diagnosing an isolated CMC joint dislocation on the initial examination is difficult as it may be obscured by diffuse swelling of the hand [4]. According to a study by Henderson and Arafa in 1987, it was found that out of 21 patients with carpometacarpal dislocations, the diagnosis was missed in 15 cases in the accident and emergency department [5]. The anteroposterior and oblique views of the hand may also prove treacherous to the untrained eye, as the overlapping bones can obscure the pathology [6].

Iqbal et al. describe the Indian salutation test which may prove useful for acute dorsal dislocations of the ulnar four fingers [7]. On X-ray, one can look for the loss of the parallel appearance of opposing margins of the CMC joint and for overlapping at the CMC joint. Horneff et al. describe drawing metacarpal cascade lines to look for bony alignment and as mentioned in this case, a lateral view can be taken as well [8].

The longitudinal and transverse arches of the hand are disrupted following a CMC dislocation [9]. This can lead to decreased range of movement and poor grip. In order to restore the functional ability, reduction of the CMC joint is necessary.

Harwin et al. describe a case of isolated dislocation of the second and third CMC joints which required K-wire fixation to maintain the reduction [10]. Ho et al. reported a case of isolated dorsal dislocation of the second CMC joint, which was not amenable to closed reduction and hence, open reduction was performed where the extensor carpi radialis brevis was found to be interposing between the base of the second metacarpal and the trapezoid [2].

With closed reduction fails, open reduction is done keeping in mind the possibility of extensor tendon interposition. This is followed by a transfixation by K-wire to maintain the reduction.

## Conclusions

Isolated CMC joint dislocations can be easily missed, both clinically and radiologically. In such cases, a lateral X-ray view or CT can be useful. Closed reduction must be attempted as soon as possible or, as in our instance, open reduction along with K-wire transfixation may be done. A regular follow-up is crucial to the patient’s rehabilitation after both closed, as well as open reduction. Vigilance in identifying and managing such rare hand injuries is essential for optimal patient outcomes.

## References

[1] Sharma AK, John JT (2005). Unusual case of carpometacarpal dislocation of all the four fingers of ulnar side of hand. Med J Armed Forces India.

[2] Ho PK, Choban SJ, Eshman SJ, Dupuy TE (1987). Complex dorsal dislocation of the second carpometacarpal joint. J Hand Surg Am.

[3] Nakamura K, Patterson RM, Viegas SF (2001). The ligament and skeletal anatomy of the second through fifth carpometacarpal joints and adjacent structures. J Hand Surg Am.

[4] Telich-Tarriba JE, Guevara-Valmaña OI, Navarro-Barquín DF, Victor-Baldin A (2020). Carpometacarpal joint dislocations: management and long-term outcomes at a specialized hand surgery center in Latin America. Plast Surg (Oakv).

[5] Gibson MF, Clancy MJ (1990). Carpometacarpal dislocation: an unusual complex injury of the hand. Arch Emerg Med.

[6] Barskaya A, Lowy R, Acosta N (2023). Man with left hand pain. J Am Coll Emerg Physicians Open.

[7] Iqbal MJ, Saleemi A (2003). Indian salutation test in acute dorsal carpometacarpal joint dislocation of the ulnar four fingers. Am J Emerg Med.

[8] Horneff JG 3rd, Park MJ, Steinberg DR (2013). Acute closed dislocation of the second through fourth carpometacarpal joints: satisfactory treatment with closed reduction and immobilization. Hand (N Y).

[9] Woon CY, Chong KC, Low CO (2006). Carpometacarpal joint dislocations of the index to small finger: three cases and a review of the literature. Injury Extra.

[10] Harwin SF, Fox JM, Sedlin ED (1975). Volar dislocation of the bases of the second and third metacarpals. A case report. J Bone Joint Surg Am.

